# Exploring Microtubule-Dependent Cellulose-Synthase-Complex Movement with High Precision Particle Tracking

**DOI:** 10.3390/plants7030053

**Published:** 2018-07-04

**Authors:** Marcus Woodley, Adam Mulvihill, Miki Fujita, Geoffrey O. Wasteneys

**Affiliations:** Department of Botany, University of British Columbia, Vancouver, V6T 1Z4 BC, Canada; mwoodley94@gmail.com (M.W.); adm0301@gmail.com (A.M.); miki.fujita@botany.ubc.ca (M.F.)

**Keywords:** cellulose-synthase-complex, microtubule, growth anisotropy, particle tracking, TIRF

## Abstract

Cellulose synthesis at the plasma membrane is a critical process in plant growth and development. The displacement of cellulose synthase complexes (CSCs) by the rigid cellulose polymers they produce is a measure of enzyme activity. Connections between cortical microtubules and CSCs have been identified but it remains unclear how these affect CSC displacement speed. In this study, we applied a high throughput automated particle tracking method using near-total internal reflection fluorescence microscopy to measure the speed of CSCs. We found CSC speeds did not vary according to their proximity to microtubules, and that inhibiting microtubule polymerization could have opposite effects on CSC speed, depending on the nature of inhibition. While CSC speed increased in the temperature-sensitive *mor1-1* mutant, it decreased after treatment with the drug oryzalin. Moreover, introducing the *mor1-1* mutation into the CesA1 mutant *any1* increased CSC speed, suggesting that microtubule dynamics affect CSC speed by a mechanism other than Cellulose Synthase A (CesA) catalytic activity. CSC speed varied widely in a range of mutants with reduced growth anisotropy, indicating that the relationship between CSC speed and anisotropy is complex. We conclude that microtubules affect CSC speed by finely tuned mechanisms that are independent of their physical association with CSCs.

## 1. Introduction

The plant cell wall is essential for the proper growth and morphology. Cellulose acts as the primary tension-bearing component within the complex cell wall structure. In order to properly regulate cell growth and anisotropy, plant cells must balance their internal turgor pressure via modification of the cell wall [1]. If cellulose is not properly synthesized, this leaves the cell wall compromised, meaning plants may be unable to properly manage their own turgor pressure, which can cause deleterious effects such as severe dwarf phenotypes and radial swelling. The process of cellulose synthesis has been extensively investigated but currently there are many unanswered questions. For example, although the movement of cellulose synthase complexes (CSCs) at the plasma membrane can be attributed to displacement in accordance with the amount of cellulose that is synthesized by each complex, whether this varies according to the proximity of CSCs to cortical microtubules remains unclear. Similarly, although previous studies have shown that the loss of growth anisotropy in some mutants is accompanied by shifts in both the CSC displacement velocity and cellulose crystallinity [2,3,4], the molecular mechanisms controlling these processes remain obscure.

To date, monitoring cellulose synthesis in vivo by the movement of fluorescently labelled CSCs is most often measured using “Kymograph Analysis” [5]. From a time series of fluorescent CSC images captured with a fluorescence microscope, a linear row of pixels of the particles of interest are arranged in series and plotted against acquisition time. Unidirectional movement of a CSC will display as a diagonal line on the kymograph, the slope of which can be measured to calculate its velocity.

Kymograph analysis has proved suitable for detecting large changes in the behaviour of CSCs between different mutants and treatments [2,3,5,6,7,8,9]. It is not, however, without drawbacks. Obtaining an adequate sample size is time consuming because each particle must be individually analyzed. Kymograph analysis is also only able to measure the displacement of particles that exhibit consistent linear trajectories. By measuring the object’s change in position, kymographs do not take into account any deviations from a straight path that the object might take. It is also subjective because each kymograph needs to be interpreted separately by human operators, which can easily influence results via unintentional bias [10,11]. Although blinding is generally presented as an effective solution to avoid unintentional bias [10], it is not always practical because genotypes can usually be easily distinguished by features such as cell shape when measuring kymographs.

In this study, we explored the use of particle tracking as an alternative to kymograph analysis to address several questions on microtubule-dependent CSC movement. Particle tracking has multiple advantages, including the ability to detect non-linear trajectories and the capacity to process a larger sample size in an unbiased manner. We illustrate the effectiveness of particle tracking for assessing genotype-specific differences in CSC movement and for evaluating with unprecedented accuracy how proximity to cortical microtubules affects CSC speed. Our data provide new insights, particularly with respect to the microtubule-dependent behaviour of CSCs.

## 2. Results

### 2.1. Automated Particle Tracking Quickly and Accurately Measures Fluorescent Protein Distribution and Dynamics

To improve the gathering of data related to the movement of CSCs as a proxy for cellulose synthesis rates, we explored the particle-tracking features of the bioimaging analysis platform ICY [12]. By automating the data acquisition and analysis process, we were able to rapidly collect larger data sets and avoid the possibility of subjecting our data to unintentional bias [11]. This method also takes into account any non-linear CSC pathways, and thus our outputs are described as CSC speed, in contrast to previous studies where we, and others, have described CSC displacement in terms of velocity, reflecting their vectoral nature. Hereafter, we describe kymograph data as velocity, and particle tracking data as speed.

Previous studies have shown that CSC movement is faster on average in the temperature-sensitive *mor1-1* mutant relative to wild type at its restrictive temperature [2]. To compare kymograph and particle tracking, time-lapse images were collected on a Zeiss microscope equipped with a total internal reflectance fluorescence (TIRF) module for 3-day-old dark-grown hypocotyls of both wild-type and *mor1-1* seedlings expressing YFP-CesA6 and the tubulin reporter mRFP-TUB6. The use of the near-TIRF setting (also known as variable angle epifluorescence) greatly eliminated the signal interference from cytoplasmic trafficking of YFP-CesA6 contained in cytoplasmic compartments. When using spinning disk confocal microscopy, this problem greatly restricts the regions of interest that can be used for time-lapse imaging of CSCs. In addition, since temperature has been shown to have a major effect on CSC movement [2], we used a temperature-controlled stage to maintain temperature at 29 °C, which is both the restrictive temperature for the *mor1-1* mutant but also a temperature at which CSCs are relatively easy to track [2]. The resultant image datasets were then processed to calculate the velocity and speed of fluorescent CSCs using both kymograph and particle tracking respectively. We generated the line we refer to hereafter as *mor1-1* by crossing a *prc1-1* (*CesA6* null) mutant expressing YFP-CesA6 and mRFP-TUB6, with *mor1-1* and segregating *mor1-1* homozygotes in the F2 generation. The wild-type line was segregated from F2 progeny of this line that were azygous for *mor1-1* [2].

**Figure 1:**
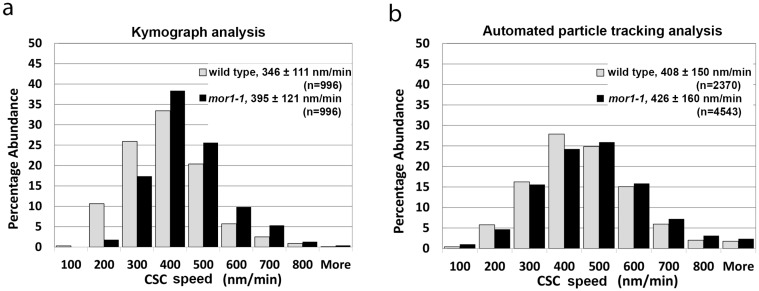
Automated particle tracking quickly and accurately measures fluorescent protein distribution and dynamics. (**a**,**b**) Histogram of cellulose synthase complex (CSC) velocity distribution in wild type and in *mor1-1* recorded at 29 °C using kymograph analysis (**a**), and automated particle-tracking analysis (**b**). CSC velocity and speeds are significantly greater in *mor1-1* compared to wild type (*p* < 0.0001) for kymograph and particle-tracking analysis respectively. Values are means ± SD. See also Movies S1 and S2.

According to kymograph analysis, we recorded a mean CSC velocity of 346 nm/min for wild-type seedlings (*n* = 996, SD = 110) and 395 nm/min (*n* = 996, SD = 121) for *mor1-1* (Figure 1a, Movie S1 and S2). By comparison, automated particle tracking determined CSC speeds of 408 nm/min (*n* = 2370, SD = 150) for the wild type and 426 nm/min for *mor1-1* (*n* = 4543, SD = 160) (Figure 1b). Both datasets were consistent in showing a statistically significant increase in the movement of CSCs in *mor1-1* relative to wild type, as previously reported [2]. We also noted that data sets analyzed with particle tracking consistently generated higher mean CSC speeds than those measured with kymograph analysis, and that these differences were statistically significant.

### 2.2. Cellulose Synthase Complex Speed Is Not Correlated with Proximity to Microtubules

The *mor1-1* mutant has a conditional phenotype when grown at 29 °C, which greatly reduces the rate of microtubule growth and shrinkage [13], and disrupts the parallel organization of microtubules [14]. Past research using coincidence analysis reported that approximately 60% of CSCs in the wild type track in close association with microtubules whereas this association decreases to 48% in *mor1-1* [2]. Based on this difference, we hypothesized that CSCs that are closely associated with microtubules move more slowly than those that track in regions not associated with microtubules.

**Figure 2:**
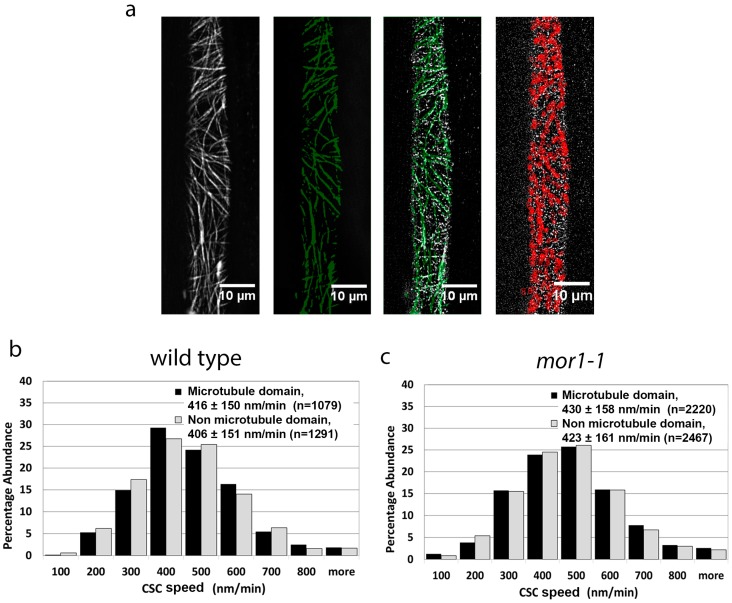
Cellulose synthase complex speed is not correlated with proximity to microtubules. (**a**) Particle tracking steps for CSCs in microtubule domains. Projections of 5-min time-lapse microtubule images (first panel) were used to define the microtubule domains by thresholding (green in second panel). The microtubule domain image was overlaid with CSC time-lapse images. The third panel is a single frame showing the overlay of microtubule domains (green) and YFP-CesA6 particles (white). Using spot detection plugins, CSCs were filtered from completely within the regions of interest, thus allowing the tracking of particles that remain completely within microtubule domains, as shown in the fourth panel as red. (**b**,**c**) Comparison of CSC velocity in microtubule domains versus non-microtubule domains in wild-type (**b**) and *mor1-1* seedlings (**c**). Values are means ± SD. There was no significant difference in CSC velocity between microtubule and non-microtubule domains in both wild type and *mor1-1*. Samples were incubated at 29 °C.

The superior resolution of the variable angle epifluorescence imaging provided by the TIRF imaging system enabled us to use the particle tracking software to compare the speed of CSCs tracking in close proximity to microtubules with those that do not. The steps used to compare CSC movement within and outside microtubule domains are shown in Figure 2a. Using the same image datasets used in Figure 1a,b, we performed particle tracking of microtubule-associating and non-microtubule-associating CSCs (Figure 2b,c). For both the wild-type (Figure 2b) and *mor1-1* (Figure 2c) seedlings, we measured no significant difference in the speed of CSCs associating with microtubules (means = 416 nm/min for wild type, 430 nm/min for *mor1-1*) and those not associating with microtubules (means = 406 nm/min for wild type, 423 nm/min for *mor1-1*). These data do not support the hypothesis that microtubule-associated CSCs move more slowly, and specifically demonstrate that the reduced overlap of CSCs with microtubules in *mor1-1* at its restrictive temperature does not account for the more rapid movement of CSCs.

### 2.3. The Microtubule-Destabilizing Drug Oryzalin Reduces CSC Speed

**Figure 3:**
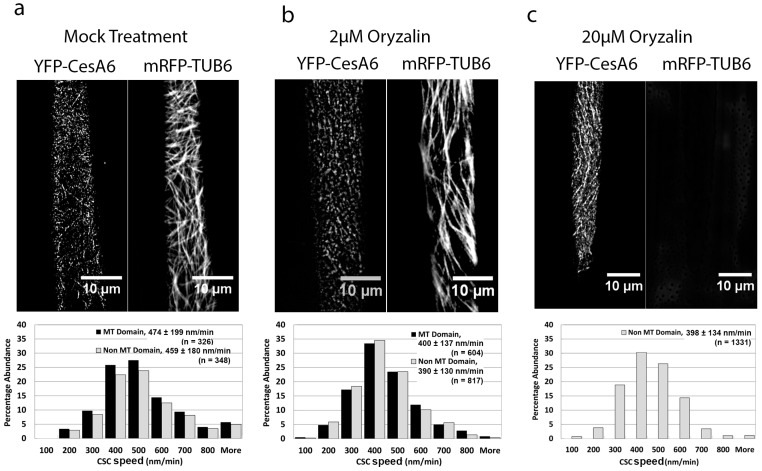
The microtubule-destabilizing drug oryzalin reduces CSC speed. (**a**–**c**) Projections of 5-min time-lapse images of YFP-CesA6 and mRFP-TUB6, and CSC speed distribution in microtubule and non-microtubule domains in wild type after 3 h treatments with 0.1% DMSO (mock) (**a**), 2 µM oryzalin/0.1% DMSO (**b**), and 20 µM oryzalin/0.1% DMSO (**c**). Values are means ± SD. CSC speed in oryzalin-treated seedlings was significantly reduced compared to that of mock treatment (*p* < 0.0001). There was no significant difference in mean CSC speed between microtubule and non-microtubule domains in mock and oryzalin treatments. All samples were incubated at 29 °C. See also Movies S3–S5.

To determine if the increased speed of CSCs in *mor1-1* at its restrictive temperature is a function of the reduced microtubule polymer mass, we treated wild-type seedlings with a mild dose of the microtubule-destabilizing drug oryzalin. Based on the *mor1-1* phenotype, we hypothesized that the partial depolymerization of microtubules should result in an increased CSC speed. We first determined that a 3 h treatment with 2 μM oryzalin caused a partial reduction in microtubule polymers that was roughly similar to that shown in *mor1-1*. In contrast to our prediction, however, the 2 μM oryzalin treatment caused a significant reduction in the speed of CSCs compared to the mock (0.1% DMSO) treatment (Figure 3a,b, Movies S3 and S4). We also detected no significant difference in the speed of CSCs tracking in association with microtubules and those not associated with microtubules for both oryzalin- or mock-treated samples (Figure 3a,b). We next exposed seedlings to 20 μM oryzalin, which after 3 h, completely disassembled microtubules (Figure 3c, Movie S5). In the complete absence of microtubules, the mean CSC speed was 397 nm/min, which was significantly slower than the mock treatment but not different from the 2 μM treatment. In conclusion, the reduction or elimination of microtubule polymers by oryzalin treatment caused a reduction in CSC speed, in contrast to the significant increase that occurs in the *mor1-1* mutant.

### 2.4. The mor1-1 Mutation Increases CSC Speed through a Mechanism That Is Independent of the CSC Catalytic Activity

To understand the cause of the increased CSC speed in the *mor1-1* mutant we considered the possibility that this involves a change in the enzymatic activity of the CSCs. A previous study demonstrated that the CesA1 missense mutant *any1* has reduced CSC velocity (as measured by kymograph analysis) and wall crystallinity (as measured by X-ray diffraction) but that the overall production of cellulose is unaffected [2]. Based on this, we proposed two alternative hypotheses. According to the first hypothesis, if the increased CSC speed in *mor1-1* is caused by an increase in the catalytic activity of the CSCs, the *any1mor1-1* CSC speed should be similar to that of the *any1* single mutant since it is unlikely that a defect in catalytic activity can be ameliorated. According to the second hypothesis, if factors other than enzymatic activity of CesA proteins are responsible for the increased CSC speed in *mor1-1*, the CSC speed in *any1mor1-1* double mutants should be increased relative to that of the *any1* single mutant.

**Figure 4:**
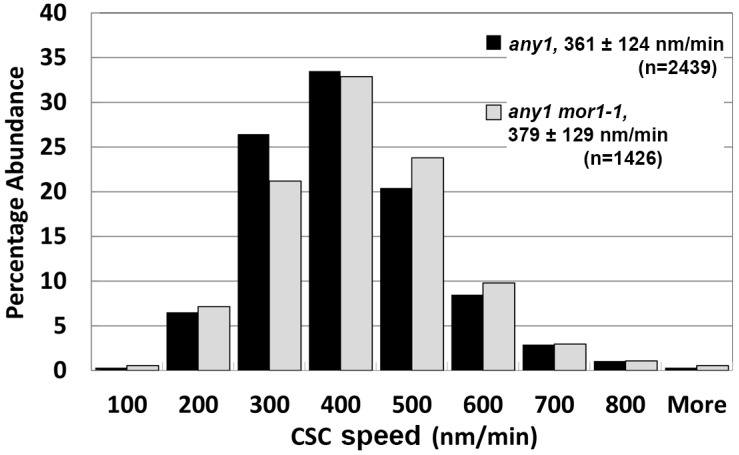
The *mor1-1* mutation increases CSC speed through a mechanism that is independent of the CSC catalytic activity. CSC speed distribution in *any1* single mutant and the *any1mor1-1* double mutant at 29 °C.

To test these hypotheses, we crossed the *any1* mutation into our experimental lines to generate a *mor1-1*, *any1*, *prc1* triple mutant carrying the YFP-CesA6 reporter. According to particle tracking analysis, the mean CSC speed in *any1* was 361 nm/min (*n* = 2439) while in *any1 mor1-1* it was significantly higher at 379 nm/min (*n* = 1426) (Figure 4). From this, we conclude that the increased CSC speed in *mor1-1* is caused by factors other than CSC enzymatic activity.

### 2.5. Moderate CSC Speed Is Required but Not Sufficient for Cell Growth Anisotropy

**Figure 5:**
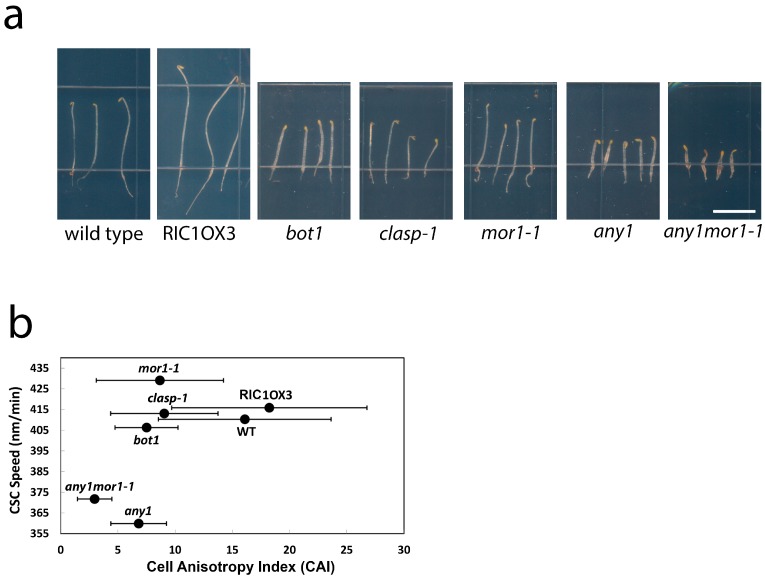
Moderate CSC speed is required for cell growth anisotropy. (**a**) Images of 3-day-old dark-grown seedlings at 29 °C used in anisotropy measurements; (**b**) CSC speed and cell anisotropy index (CAI) of the various analyzed genotypes were plotted. CAI was calculated as the ratio of the length divided by width of the hypocotyl epidermal cells. Wild-type CAI = 16.1 ± 7.6 (*n* = 60). *mor1-1* CAI = 8.6 ± 5.6 (*n* = 49). *any1* CAI = 6.8 ± 2.5 (*n* = 82). *bot1* CAI = 7.5 ± 2.8 (*n* = 61). RIC1OX3 CAI = 18.2 ± 10.4 (*n* = 92). *any1mor1-1* CAI = 3.3 ± 1.5 (*n* = 181). *clasp-1* CAI = 9.0 ± 4.7 (*n* = 99). CSC speed was measured by tracking YFP-CesA6 particles except in *clasp-1*, which used GFP-CesA3.

Previous work showed that in *mor1-1*, the increased velocity of CSCs relative to wild type is correlated with increased cellulose crystallinity and a loss of growth anisotropy [2]. This finding suggests that in order to maintain anisotropy under rapid growth conditions, wild-type cells constrain CSC speed to generate more amorphous cellulose microfibrils, which are more likely to establish cross links with surrounding matrix polysaccharides [2,15]. To explore the potential relationship between CSC speed, cellulose crystallinity, and growth anisotropy, we measured the length and diameter of the epidermal cells and calculated the ratio of length over diameter as a cell anisotropy index (CAI) for the wild type and a selection of mutants with altered growth anisotropy (Figure 5a). The RIC1OX3 line [16] has increased anisotropy [2] as a result of overexpressing the RIC1 microtubule-associated protein, whereas *bot1* [17], *mor1-1* [14], and *clasp-1* [18] have reduced growth anisotropy as a result of loss of function mutations in microtubule-associated proteins. We also included the *any1* mutant of CesA1 [3], which has a reduced growth anisotropy phenotype (Figure 5a) as well as the *mor1-1 any1* double mutant. Plotting the CAI against mean CSC speed measured at 29 °C resulted in no clear correlation (Figure 5b). The CSC speeds for the *clasp-1* and *bot1* mutants, for example, were very similar to those recorded for the wild type and RIC1OX3, yet the CAI varied from 7.5 and 9 in *bot1* and *clasp-1*, respectively, to 16.1 and 18.2 in wild type and RIC1OX3, respectively. Notably, *any1* (360 nm/min) and *mor1-1* (430 nm/min), despite having the most divergent CSC speeds of all genotypes tested, have very similar CAIs. This indicates that excessive speed or low speed can be equally detrimental for growth anisotropy. The relationship, however, is clearly complex. In the *any1 mor1-1* double mutant, for example, despite a CSC mean speed that is intermediate between the *any1* and *mor1-1* single mutants, the CAI is lower than either *any1* or *mor1-1*. In conclusion, while moderate CSC speed appears to be essential for sustaining growth anisotropy, there are other factors that also influence this process.

## 3. Discussion

In this study, a near-TIRF microscopy and a high-throughput particle-tracking method enabled us to determine that CSC speed at the plasma membrane is independent of their association with cortical microtubules. This finding is important because it indicates that cortical microtubules, while clearly essential for defining the physico-chemical properties of cellulose and the material anisotropy of expanding cell walls, do not define spatial heterogeneities, or domains, that specifically affect the speed at which cellulose is produced by CSCs. Instead, our data suggest that perturbations to microtubule dynamics alter the global behaviour of CSCs.

The results of our study should help to elucidate the specific function of proteins such as CSI1/POM2 that can link CSC to microtubules [8,9,19]. Despite the presence of these linkers, a substantial proportion of CSCs track independently of microtubules [5], and the proportion can vary considerably between cell types or after changes in microtubule organization [2,20]. Interestingly, changes in the degree of CSC-microtubule association have been correlated with changes in CSC motility. In experimentally induced stomatal closure [20], or in the *mor1-1* mutant under non-permissive conditions [2], CSC-microtubule coincidence is reduced while the CSC speed increases. In contrast, in the *csi1-3* mutant, there is a reduction in both the CSC-microtubule coincidence and CSC velocity [19]. With our near-TIRF particle tracking method we found no significant differences between microtubule-associated and microtubule-independent CSCs in terms of mean speed and range of speed.

Our analysis of a range of growth anisotropy mutants associated with altered microtubule dynamics and organization determined that shifts in CSC speed are genotype-dependent. A strong deviation from wild-type CSC speed was only found in the *mor1-1* mutant, which, compared to the other genotypes, has severely impaired microtubule plus end dynamics [13]. Similarly, treatment with the microtubule-destabilizing drug, oryzalin, altered CSC speed. It is intriguing, however, that the loss of microtubule polymers in *mor1-1* and oryzalin treatments have opposite effects on CSC speed. Through particle-tracking analysis, we demonstrated that a reduction in microtubule polymer mass was associated with increased CSC speed in *mor1-1* and decreased speed in oryzalin treatments, consistent with previous kymograph-based studies [2,19]. From this, we can conclude that CSC activity, and hence displacement speed, is under tight control by as yet unidentified mechanisms associated with microtubule dynamics. Moreover, deviations in opposite directions from a moderate speed (too fast in *mor1-1*, too slow in oryzalin) are clearly detrimental in terms of optimal wall mechanical properties and growth anisotropy.

While it remains unclear how *mor1-1′*s defective microtubule dynamics stimulate more rapid CSC movement, by exploiting the attributes of the CesA1 point mutant *any1*, we were able to rule out increased CSC catalytic activity as the cause. Both *mor1-1* and *any1* have compromised growth anisotropy without measurable losses of cellulose production, but both CSC speed and cellulose crystallinity are low in *any1* [21] and both are high in *mor1-1* [2]. The significantly higher CSC speeds in *any1 mor1-1* double mutant seedlings compared to the *any1* single mutant suggest that the defect in microtubule dynamics in the *mor1-1* mutant is affecting some factor other than CesA activity. One possibility that we are currently investigating is whether microtubule disruption in *mor1-1* leads to changes in the fluidity of the plasma membrane. Higher than optimal fluidity would allow less resistance for displacing CSCs resulting in faster mean CSC speeds, higher crystallinity, and reduced growth anisotropy.

Cellulose synthesis must be carefully balanced through CSC speed and abundance at the plasma membrane, along with cellulose crystallinity and the degree of polymerization. By improving the ability to record unobstructed CSC movements with temperature-controlled near-TIRF microscopy, and utilizing the high-throughput and unbiased aspects of automated particle tracking, we have answered several key questions about the relationship between CSC movement and microtubules. This technique holds much promise for further investigations of cellulose synthesis and the maintenance of growth anisotropy under a wide range of genetic and environmental conditions.

## 4. Materials and Methods

### 4.1. Plant Materials and Growth Conditions

Arabidopsis plants expressing YFP-CesA6 [5] and mRFP-TUB6 [21] in the *prc1-1*, *cesa6* null, mutant background [2] were crossed with *botero1* (*bot1*) [17] provided by Dr. Herman Höfte (Centre de Versailles-Gringon, INRA, France), and RIC1-OX3 [16] provided by Dr. Zhen-Biao Yang (Center for Plant Cell Biology, University of California at Riverside, CA). F3 and F4 seedlings were selected for the homozygous *prc1-1*, YFP-CesA6 and respective homozygous genotyping background. GFP-CesA3 plants [6] provided by Dr. Herman Höfte were crossed with *clasp-1* [18].

Plants expressing YFP-CesA6 in the *mor1-1/prc1-1* double mutant background [2] were crossed with plants expressing YFP-CesA6 in *any1/prc1-1* [3] to generate an *any1/mor1-1/prc1-1* triple mutant expressing YFP-CesA6.

Plants were cultured on Hoagland’s medium in 1.2% agar in vertically held plates covered with aluminum foil. Seeds were incubated at 4 °C for 4 days, then germinated at 21 °C. Seedlings were grown for 2 days at 21 °C and then 1 day at 29 °C prior to imaging.

For the cell anisotropy measurements, seeds were exposed to light for 3 h prior to a 3-day cold treatment, then germinated and grown at 29 °C.

### 4.2. Near-TIRF Imaging of CesAs

Three-day-old dark-grown seedlings were mounted in a hormone solution containing 10 μM gibberellic acid and 0.5 μM indole acetic acid as described previously [22]. The mounting solution and all tools were heated to 29 °C prior to imaging. Seedlings were allowed approximately 10 min to acclimate before imaging began.

The TIRF microscopy system consisted of a Zeiss Observer.Z1 microscope, TIRF 3.1, equipped with a Q Imaging Rolera EMCCD camera and Hamamatsu Orca Flash sCMOS camera, and a 63X/1.46 Oil Korr TIRF objective lens, controlled by Zen software (Zeiss). A 488 nm excitation laser and 525/50 nm emission filter were used for YFP and GFP imaging. A 561 nm excitation laser and a 601/25 nm emission filter were used for RFP imaging. Samples were incubated at 29 °C for 24 h prior to imaging. During imaging, the temperature of both the objective lens and stage was maintained at 29 °C. Five-minute time-lapse videos were recorded in 10-s increments closest to the elongation zone of the hypocotyl.

### 4.3. Image Processing

All image processing was done using ImageJ software. Brightness and contrast were linearly adjusted to highlight the detail as best as possible. The MultiStackReg [23] plugin was used to correct for sample drift over the course of imaging. Next the WalkingAverage plugin was used to minimize unwanted background noise. Stacked average images of the time series were created for YFP-CesA6 and mRFP-TUB6 channels in order to easily track the entire path of CSCs and microtubules. Clear CSC paths were highlighted and the MultiKymograph tool was then used to create a kymograph of the highlighted path.

### 4.4. Particle Tracking

All particle tracking was done using ICY bioimaging analysis software. Microtubule domains were highlighted using the ICY threshold function in order to create specific microtubule-domain and non-microtubule-domain regions of interest. When analyzing the effects of microtubule domains on CSC speed, particles must remain either in or out of the domain throughout the entire imaging period. Individual particles were tracked using the spot detector and spot tracking plugin. During particle tracking all tracks maintained continuous tracking over their durations, with no skipped frames. Tracks with durations of 30 s or less were removed from the data to be analyzed.

### 4.5. Cell Anisotropy Measurement

Hypocotyl epidermal cells of 3-day dark-grown seedlings were imaged using a Zeiss Axioplan2 microscope. The cell length was divided by cell width to calculate the anisotropy index.

### 4.6. Statistical Analysis

All data were analyzed using graphpad PRISM statistical analysis software. A one-way ANOVA test was done along with a Kruskal–Wallis post-hoc test for non-parametric data. For the data showing abnormal distribution, a Wilcoxon rank sum test was used to determine a significant difference.

## Supplementary Materials

Supplementary materials can be found at http://www.mdpi.com/2223-7747/7/3/53/s1. Movies S1–S5. Distribution and movement of YFP-CesA6 (green) and mRFP-TUB6 (magenta) in wild type (Movie S1), *mor1-1* (Movie S2), 0.1% DMSO (mock) after 3 h (Movie S3), 2 μM oryzalin (Movie S4) and 20 μM oryzalin (Movie S5). Scale bars show 5 μm. Movies play 100 times more quickly than real time.
